# Robust Segmentation of Planar and Linear Features of Terrestrial Laser Scanner Point Clouds Acquired from Construction Sites

**DOI:** 10.3390/s18030819

**Published:** 2018-03-08

**Authors:** Reza Maalek, Derek D Lichti, Janaka Y Ruwanpura

**Affiliations:** 1 Department of Civil Engineering, University of Calgary, Calgary, AB T2N 1N4, Canada; janaka@ucalgary.ca; 2 Department of Geomatics Engineering, University of Calgary, Calgary, AB T2N 1N4, Canada; ddlichti@ucalgary.ca

**Keywords:** TLS/LiDAR point clouds, robust statistics, minimum covariance determinant (MCD), robust principal components analysis (PCA), robust planar and linear segmentation

## Abstract

Automated segmentation of planar and linear features of point clouds acquired from construction sites is essential for the automatic extraction of building construction elements such as columns, beams and slabs. However, many planar and linear segmentation methods use scene-dependent similarity thresholds that may not provide generalizable solutions for all environments. In addition, outliers exist in construction site point clouds due to data artefacts caused by moving objects, occlusions and dust. To address these concerns, a novel method for robust classification and segmentation of planar and linear features is proposed. First, coplanar and collinear points are classified through a robust principal components analysis procedure. The classified points are then grouped using a new robust clustering method, the robust complete linkage method. A robust method is also proposed to extract the points of flat-slab floors and/or ceilings independent of the aforementioned stages to improve computational efficiency. The applicability of the proposed method is evaluated in eight datasets acquired from a complex laboratory environment and two construction sites at the University of Calgary. The precision, recall, and accuracy of the segmentation at both construction sites were 96.8%, 97.7% and 95%, respectively. These results demonstrate the suitability of the proposed method for robust segmentation of planar and linear features of contaminated datasets, such as those collected from construction sites.

## 1. Introduction

Construction project progress monitoring and dimensional compliance control are essential to allow decision makers to identify discrepancies between the planned and the as-built states of a project and take timely measures where required. In practice, monitoring is performed manually, which is a time consuming, error-prone and labour-intensive task, particularly for large scale projects [1]. Hence, to reduce the time and cost associated with such manual approaches while fostering practicality, only a limited amount and/or frequency of onsite data is collected, which diminishes the ability of the project proponents for timely identification of delays, rework, and cost overruns.

In addition, the reliable determination of a project’s performance is highly dependent on the accuracy of the data collected during the monitoring process. Currently, site supervisory personnel spend 30–50% of their time manually inspecting and controlling the quality of the manually-collected data [2]. Reduction of this time by means of a novel approach to onsite data collection and analysis will allow more time to be allocated to improving vital construction related concerns such as safety [3], as well as workforce productivity and communications [4].

To help overcome the limitations associated with current manual monitoring practices, the application of terrestrial laser scanning (TLS) to acquire 3D point clouds of construction site environments has been growing markedly. Since the most generic building elements are constructed from planar (columns, beams and slabs) and linear (reinforcement bar) features [5], the automatic detection of planar and linear features from TLS point clouds is the first step towards automating the monitoring and control process. The industry, however, still lacks a comprehensive solution to the automated processing of raw TLS point clouds of planar and linear features for the following reasons:

1. Outliers exist in construction site point clouds due to data artefacts, occlusions and dust.
2. Many object-based recognition models depend on the 3D/4D planned building information model (BIM), which is neither readily available nor accurate, and the as-built is not necessarily constructed to plan. The compliance of the as-built to the planned specifications, must in fact be checked through the monitoring and control process; hence, a monitoring and control process solely reliant on the details of the planned is not desirable.
3. Most planar and linear classification and segmentation algorithms use inconsistent and subjectively-defined thresholds (see Section 2.2.2), which change from one dataset to another, and hence, are not generalizable for every environment.

To address these limitations, this manuscript reports on the development of a novel robust point cloud processing method with particular emphasis on planar and linear feature classification and segmentation using only the geometric primitives.

## 2. Literature Review

### 2.1. TLS in Construction Management

#### 2.1.1. Application of TLS to Measure Key Performance Indicators

Key Performance Indicators (KPIs) include onsite data collected to measure the performance of a project. To measure the schedule performance of a project, up-to-date knowledge of the percentage of activities completed is required [6]. To measure this KPI, however, the as-built scope of work for each activity must be accurately determined, which is not feasible using traditional data collection practices [2].

To evaluate dimensional conformity, surface flatness/levelness and the length of structural elements are the most commonly collected KPIs [7]. Depending on the task in hand, the availability of skilled personnel and budget, onsite data are collected using instruments such as measuring tapes, carpenter’s levels, straight edges and total stations. Therefore, only limited information can be derived from the current data collection practices, and only a portion of the site can be practically monitored. This is more evident in the estimation of surface flatness or levelness. Using traditional instruments, only a fraction of the surfaces can be controlled for potential defects [8].

TLS provides 3D point clouds of surrounding surfaces, and hence can be used as a means of automatically measuring the aforementioned KPIs. However, due to the many objects present on construction sites, the manual interpretation of the KPIs from dense point clouds is tedious and impractical [9,10]. Therefore, to utilize TLS to its fullest capacity, the automatic extraction of construction site elements from point clouds has been the topic of research in recent years.

#### 2.1.2. State-of-the-Art in Automated TLS Object Extraction

Current research in construction management is devoted to the automatic extraction of the “scope of the work performed” for each activity from the acquired TLS point clouds. To this end, many research studies propose the superimposition of the planned 4D BIM and the 3D points clouds captured from TLS [11]. This method is commonly referred to as “Scan vs. BIM” in current literature [12]. The “Scan vs. BIM” approach is not, however, reliable when the actual locations of construction elements differ from those presented in the plan [13] or the issued-for-construction (IFC) BIM is not available with sufficient detail. “Scan vs. BIM” approaches also aim at converting the BIM to the same level of detail of the acquired point cloud to allow their comparison. Hence, to provide a comprehensive solution to the automated monitoring and control processes while minimizing the dependency of the object extraction on the details of the planned BIM, a more fundamentally sound approach is to extract common structural elements using only their “geometric primitives”. For a comprehensive over-view of the state of research in construction management, the reader is encouraged to review [12,14].

The initial step to automatically extract features from point clouds is labelling and grouping of points with similar geometrical attributes, commonly referred to as the classification and segmentation processes, respectively [5]. As mentioned, the classification and segmentation of planar and linear features from TLS point clouds acquired from construction sites is an essential step to identifying the most important structural elements. In the following section, the state-of-the-art research for planar and linear classification and segmentation is reviewed.

### 2.2. Automated Classification and Segmentation of Planar and Linear Features from TLS Point Clouds

To avoid confusion, from this point on, the following notions are used throughout this manuscript:

- Planar (linear) classification: The process of extracting points that locally follow a planar (linear) pattern within a point cloud dataset. In other words, points that locally follow a planar (linear) pattern are classified as planar (linear) regardless of their specific parametric equation.
- Planar (linear) segmentation: The process of grouping the classified planar (linear) points that follow a similar planar (linear) parametric equation. In other words, points that are globally on the same plane (line).

#### 2.2.1. Robust PCA-Based Point Cloud Classification

Local behaviour of points is one of the most widely utilized methods to classify planar and linear points in a point cloud dataset. There are three commonly-used methods for local planar or linear parameter estimation, namely the 3D Hough transform, random sample consensus (RANSAC), and principal components analysis (PCA). Vosselman et al. used 3D Hough transform to assign planar attributes to every point in Cartesian, which allows the determination of planar surfaces without the initial estimation of the local normal vectors [15]. However, the use of the traditional Hough transform for planar and linear classification of large datasets is computationally expensive [16,17], and the precision of the parameter estimation is a function of the size of parameter space cells [18]. It is shown by [19] that RANSAC is more computationally efficient and provides higher quality planar surface extraction compared to the traditional Hough transform.

Data artefacts caused by occlusions, moving objects, and dust cause outliers in construction site datasets. Most available planar/linear classification methods are only suited for datasets with no data contamination (i.e., no outliers). In addition, the classification of a dataset affected by outliers using classical statistical methods is highly influenced by the presence of outlier points [20,21]. Some studies [22,23,24,25,26] use RANSAC to deal with contaminated data. However, algorithms that use RANSAC perform poorly in large datasets and segmentation quality is sensitive to point density, accuracy and noise [17]. Furthermore, Nurannabi et al. showed that robust PCA obtains more robust results and out performs RANSAC for planar points [27].

Classical PCA is the eigenvalue decomposition of the covariance matrix of a multivariate dataset. It is used to summarize the variation of the dataset into independent (orthogonal) axes. In the case of a three-dimensional point cloud, three orthogonal axes can be determined. Many researchers have used PCA for the classification of planar surfaces [5,7,8,28,29,30,31,32,33,34,35,36,37]. The PCA is performed within a pre-defined neighbourhood of each point. For coplanar points, the variation of a noise-free dataset in the direction of the surface normal is equal to zero. For colinear points, all the variation of a noise-free dataset is summarized in one principal direction. If the pattern of the neighbourhood of a particular point forms a planar (linear) surface, the point is classified as a plane (line).

Since classical estimates of location and dispersion such as the sample mean and covariance matrix are adversely influenced by even a single outlying observation, they are not able to deal with contaminated datasets [38]. In this case, robust statistical methods can be used to estimate model parameters by reducing the effect of outliers. By definition, robust PCA is the eigenvalue decomposition of a robust covariance matrix (dispersion) estimate. There are currently two well-known multivariate dispersion estimates, namely, the minimum volume ellipsoid (MVE) and the minimum covariance determinant (MCD). The MVE is the smallest-volume ellipsoid that covers a subset of *h* data points out of a set of *n* observations. The (*n-h*) points left are the outliers of the dataset. The MCD selects *h* points out of n for which the covariance matrix has the smallest determinant. Compared to MVE, MCD is asymptotically normal [39], has a higher convergence rate [40] and is more suitable for larger sample sizes with a large percentage of data contamination [41]. Therefore, an MCD estimator is preferable for the processing of point clouds in highly occluded areas.

Two well-known MCD estimators exist: the fast-MCD [42]; and the deterministic-MCD (Det-MCD; [21]) exist. Unlike the fast-MCD, Det-MCD is permutation invariant, i.e., the outcome of the estimator is not a function of the order of the observations. This is of great importance since the reordering of the point cloud samples does not affect the result of the robust covariance estimation. In addition, the computation time of the Det-MCD is much lower than that of the fast-MCD [21]. Therefore, robust PCA through which the covariance matrix is estimated using the Det-MCD is expected to provide accurate planar/linear classification results. The advantage of using Det-MCD compared to classical covariance for planar and linear classification was demonstrated in [43].

#### 2.2.2. Planar and Linear Segmentation

Two methods are generally used to segment the classified planar/linear points, namely region growing (spatial domain) and attribute clustering (parameter domain). Region growing methods are widely implemented [5,26,27,28,29,30,31,32,44] due to their computational efficiency. Region growing methods start with the selection of an initial seed point. Points are then locally examined based on some pre-defined similarity attribute/condition to the seed point. This is typically carried out through a local neighbourhood search along with a smoothness constraint, where smoothness is defined as the angle between the normal vectors of a proximate point and the seed point. If the similarity condition is satisfied, the points are added to the initial seed point. More points are added to the region in the same manner until no other locally proximate point in the dataset satisfies the similarity condition. A new initial seed point from the remaining points in the dataset is then selected and the region is grown in the same way.

The result of the region growing segmentation may depend on seed point choice, i.e., the results are not permutation invariant and, hence, it is not considered as a robust method [45,46]. To solve this issue, [47] used the point in the dataset with the smallest residual to the locally fitted plane as the initial seed point. Reference [48] proposed to use the point with the maximum area of local plane as the initial seed point. The area of the local plane of a point was defined as the number of planar points in the neighbourhood of the point divided by the local point density. Reference [27] proposed to use the point with the least local curvature as the initial seed point. Although these studies have proposed permutation invariant methods for the selection of initial seed points, it is clear that no universally-valid criterion exists for initial seed point selection [17]. Furthermore, another common issue that has received less attention in the literature is that local points added to a region are only required to satisfy the similarity criteria with the current seed point and not to all points in the segment (or even previous seed points) [5,27]. This may result in a phenomenon commonly referred to as the chaining effect in cluster analysis when the correct similarity threshold is not utilized.

The second method for planar/linear segmentation is attribute clustering. For attribute clustering, an n-dimensional array of feature attributes is first defined. Points sharing similar attributes are grouped into the same cluster following some similarity criteria. Using attribute space clustering, it is possible to group together spatially discontinuous points. Thus, a connected components method is required, typically through a nearest neighbour search or boundary detection [16], to remove spatially disconnected points.

Many methods for clustering points in attribute space exist for planar and linear feature segmentation. In [49,50], point cloud segmentation using the k-means clustering algorithm was investigated. The k-means clustering approach, however, requires a priori knowledge of the number of clusters, which is not desirable for point cloud processing since the number of segments is generally unknown. In the work of [33], clustering of the point clouds was carried out by seeking the mode of the histogram of the estimated attributes. However, the correct identification of the mode is challenging in multivariate attribute cases [51]. In [16], a two-step segmentation method for planar and linear features is proposed. First a region growing method is deployed to identify planar/linear patches. Planar/linear patches are then merged together when the attributes of two patches are deemed similar according to a predefined threshold. Reference [36] proposes a planar segmentation method to improve computational efficiency by using only two attributes to cluster planar points instead of the typical four plane parameters by introducing two origins, similar to the linear segmentation technique presented in [43]. The distances of the classified planes to both origins are used as the similarity attributes. Points are grouped together if their attributes are within a predefined threshold, which is chosen to be two times the expected accuracy of the TLS instrument used for data collection, i.e., so chosen to theoretically cover 95% of the points. The two defined attributes, however, cannot uniquely define coplanar points. Therefore, a refining step is used that defines two new origins with respect to non-planar clusters.

Clustering methods are generally more robust than region growing since the result of the segmentation is permutation invariant. However, segmentation quality depends on the accuracy of the estimated attributes. In this manuscript, the latter problem is solved by utilizing robust PCA for the robust estimation of the desired attributes. Since the attributes in this study are accurately estimated during the classification process, compact clusters (clusters with low dispersion) are expected. In the research carried out by [52,53,54,55], the complete linkage method [56] was shown to be efficient for identifying compact clusters. This method does not require a priori knowledge about the number of clusters. In addition, it is not highly affected by outliers. However, it can break large clusters [57], resulting in over-segmentation. This is because similarity thresholds used to identify points with similar attributes are generally subjectively defined, and not precise.

The subjective definition of similarity thresholds is in fact common in both clustering and region growing methods. For instance, in [32], two locally-proximate linear (cylindrical) components are merged if the directional vectors of the two components create an angle less than 10°. Reference [44] used 5° for the exact same threshold. Reference [58] merge together two locally-proximate planar surfaces when the normal vectors create an angle less than 10°. In [27], this threshold was set to 5° and 15° for two different datasets. Reference [47] uses 15° for the same smoothness constraint. Reference [17] used 10° for one dataset and 15° for planar surface segmentation in another. Reference [26] used an angle of 4–6° for the exact same metric. Clearly, the threshold used for grouping coplanar and collinear points is currently scene dependent and subjective.

The correct selection of the aforementioned thresholds is especially important for distinguishing planar (or linear) from non-planar (or non-linear) surfaces. If the selected angular similarity threshold is large, points on non-planar (non-linear) surfaces, such as a cylinder with a large radius, may be grouped together as a plane. Another example is two linear components with different orientations that are attached via a smooth connection that may be merged into one segment [32]. This phenomenon is referred to as the chaining effect in cluster analysis.

In summary, the choice of threshold used to examine similarity is mostly subjectively defined and may result in over- or under-segmentation depending on the dataset. In this manuscript, a new iterative and robust complete linkage clustering algorithm is presented to reduce the dependency of the segmentation results on the choice of threshold and to minimize the chaining effect to only identify globally planar and linear segments (low curvature segments).

## 3. Methodology

**Figure 1:**
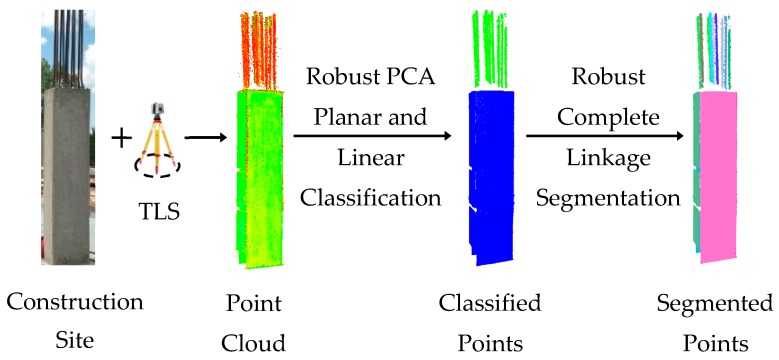
Automated as-built model generation scheme from unorganized TLS point cloud.

As mentioned, the focus of this study was to develop a generic planar and linear segmentation framework whose performance is not a function of subjectively defined attribute similarity criteria, and hence can be used in different construction site environments. The proposed framework is summarized in Figure 1. The details of each step are explained in the following subsections.

### 3.1. Robust Planar and Linear Classification

#### 3.1.1. Neighbourhood Definition

As described in Section 2.1, the behaviour of points in their local neighbourhood is used in this study to classify planar and linear points. To this end, a neighbourhood is defined around each point using the KD-tree structure. Two common methods exist to define the size of the neighbourhood of a desired point: the number of points in the proximity; and the radius of the neighbourhood. Some researchers [5,27] prefer to use a consistent number of points to minimize the impact of point density variation. However, if the dimensions of the object of desire are small and the local point density is low, unwanted points may be included in the neighbourhood, which could adversely affect the PCA classification results. To overcome this limitation, a 35 mm spherical neighbourhood size was chosen based on the dimensions of the smallest components of the structural elements that must be extracted on construction sites: reinforcement bars or rebar. 35 mm neighbourhood size was adopted since the diameters of the rebars used in regular rectangular building construction in Canada are typically smaller than 35 mm (35M rebar).

#### 3.1.2. Robust PCA

The Det-MCD estimator is used in this study for multivariate, non-parametric outlier detection due to its efficiency, accuracy and practicality. In this study, the Det-MCD estimator was independently implement; however, this estimator is available as a Matlab function through the LIBRA robust statistical toolbox [59]. The covariance matrix of the outlier-free observations is referred to as the robust covariance. Accordingly, the mean of the outlier-free observations is referred to as the robust mean, the robust centre.

Robust PCA is performed on the neighbourhood of each point to determine the local pattern/variation. Although robust PCA minimizes the influence of outliers on the eigenvalue estimation, the variations of the eigenvalues due to random measurement errors must also be quantified. This impact can theoretically be derived by variance propagation from the functional TLS positioning model and the stochastic properties of the instrumental random errors. Some research has aimed to determine the impact of point measurement errors on the principal components [60,61]. However, the exact analytical relationship between the principal components of error-free points and the principal components of the points including measurement errors when their covariance matrix contains non-zero off-diagonal elements has not been developed [60]. It can be shown using the law of variance propagation [62] that the point measurement errors have correlations (i.e., contain off-diagonal elements). In this study, Monte Carlo simulation was performed to model the expected variations of the eigenvalues of the principal components of a plane and line subject to random measurement errors. Using the simulated impacts of random measurement errors on the eigenvalues, the following classification rules were derived for the TLS instrument used in this study:

(1) $$\lambda_{min}=\min\left(\lambda_{0},\lambda_{1},\lambda_{2}\right)\to \;k_{min}=\frac{\lambda_{min}}{\lambda_{0}+\lambda_{1}+\lambda_{2}},$$

(2) $$\lambda_{max}=\max\left(\lambda_{0},\lambda_{1},\lambda_{2}\right)\to \;k_{max}=\frac{\lambda_{max}}{\lambda_{0}+\lambda_{1}+\lambda_{2}},$$

(3) $$\mathrm{Point}\;P\;\mathrm{is}:\;\{\begin{array}{l} \mathrm{Linear},\;\mathrm{if}\;k_{min}<0.04\;and\;k_{max}>0.73\\ \mathrm{Planar},\;\mathrm{if}\;k_{min}<0.03\;and\;k_{max}<0.61 \end{array},$$

where $\lambda_{0},\lambda_{1}\;\mathrm{and}\;\lambda_{2}$ are the eigenvalues of the covariance matrix of the neighbourhood of a point. The Det-MCD algorithm is then applied on the three components of the eigenvalues of the classified planar and linear points to determine the inliers of the eigenvalues. The points that follow the majority pattern of the eigenvalues are considered as the final set of classified planar/linear points.

### 3.2. Robust Planar and Linear Segmentation

The next step is to group together the classified points with similar attributes, also referred to as segmentation. To segment coplanar and collinear points, a set of attributes are selected from the results of the robust PCA classification process. These attributes must uniquely define planar and linear features.

#### 3.2.1. Attribute Definition

**Figure 2:**
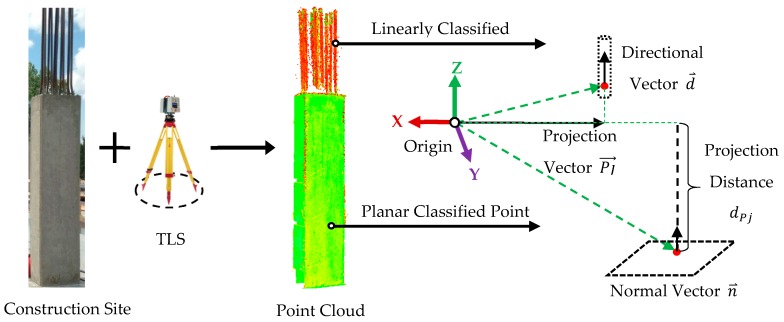
Attributes used to define linear and planar segments.

Figure 2 shows the set of attributes to uniquely define planar and linear features that are estimated during the robust PCA classification process. For each planar point, the three components of the unit normal vector ($\vec{n}$) and the distance of the plane to the origin (projection distance, $d_{Pj}$) are used. To accurately estimate the projection distance, the robust centre of the neighbourhood of a point is used instead of the Cartesian coordinates of the point. For each linear point, the three components of the unit direction vector ($\vec{d}$) and the three components of the projection vector ($\vec{Pj}$) are used. The projection vector is estimated through the application of the robust centre of the local neighbourhood of each point instead of the Cartesian coordinate of each point.

#### 3.2.2. New Iterative Complete Linkage for Point Cloud Clustering

**Figure 3:**
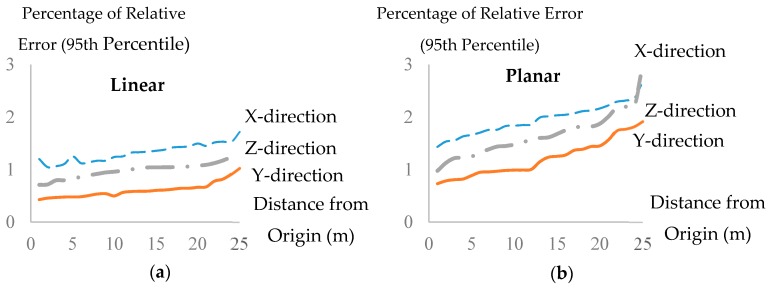
The 95th percentile of the percentage of relative error value for: (**a**) each axis of the directional and projection vector of the simulated linear points; and (**b**) each axis of the normal vector and the projection distance of the simulated planar points.

According to the complete linkage algorithm, one cluster is initially assigned to each point. The “attribute similarity” of two clusters is defined as the distance between the attributes of those clusters. Any acceptable distance metric such as Euclidian, and Mahalanobis may be used. Consequently, a pair-wise distance may be utilized for each dimension of the attribute space in case the scale or the similarity threshold for each dimension is different (the latter is implemented in this study through pair-wise thresholds expressed in Figure 3). For every cluster, the cluster with the closest attribute similarity is determined. The two clusters, say *U* and *V*, with the highest degree of attribute similarity are identified and merged together to form cluster *UV*. The attribute similarity between cluster *UV* and any cluster *W* is then calculated as:

(4) $$d_{\left(UV\right)W}=\max\left\{d_{UW},d_{VW}\right\},$$

where $d_{ij}$ is the distance between the attributes of clusters *i* and *j*. The cluster with the highest degree of similarity, say point *W*, to cluster *UV* is merged to form cluster *UVW*. The process is then continued for cluster *UVW*. The grouping is finalized when the distance measured by Equation (4) is greater than a predefined similarity threshold. The process is repeated for the remaining clusters until every cluster is visited at least once.

The complete linkage algorithm was adopted here due to its ability to merge compact clusters (clusters with low dispersion), which are expected to form due to the robust attribute estimation, adopted in this manuscript. However, it may result in over-segmentation when the similarity threshold used for segmentation is not correctly defined. To overcome this shortcoming, an initial similarity threshold is so chosen to prevent under-segmentation of the compact clusters. In other words, the similarity threshold is chosen small enough to reduce Type II errors during the segmentation process. The choice of similarity threshold is reported in Section 3.2.3.

**Figure 4:**
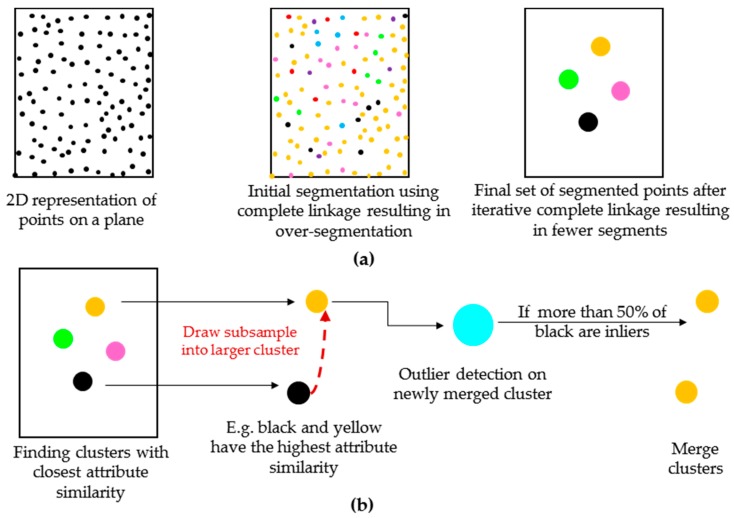
2D representation of the proposed segmentation framework (each coloured circle represents a segment): (**a**) iterative complete linkage; and (**b**) robust complete linkage.

To further reduce over-segmentation of clusters due to the choice of threshold, the “*iterative complete linkage*” process is proposed. According to this new method, the complete linkage is first performed on the estimated robust parameters to group points with similar attributes using the pre-defined similarity threshold. New attributes for the newly-merged clusters are then estimated using robust PCA (Section 3.2.1). The complete linkage algorithm is again performed for the new clusters with their new attributes to determine the prospective clusters. The process continues until the total number of clusters between two consecutive iterations remains constant (convergence criteria). The results of the iterative complete linkage method in 2D are schematically represented in Figure 4a. In Section 3.2.4, we propose a new, iterative robust method, called “*robust complete linkage*”, for merging the over-segmented clusters. As will be shown, the performance of the “*robust complete linkage*” is not a function of an arbitrarily defined similarity threshold.

#### 3.2.3. Systematic Choice of Initial Similarity Threshold

The initial similarity threshold for the complete linkage algorithm is chosen small enough to ensure over-segmentation. Since random measurement errors are expected to exist in all datasets and consequentially influence the estimated attributes, the threshold must also be large enough to at least incorporate the impact of random measurement errors. Therefore, the estimation uncertainties of the principal components (normal and directional vector) caused by random measurement errors are used to systematically quantify the initial similarity thresholds. To do so, Monte Carlo simulation is used. First, 1000 random plane (and line) orientations with 50 different distances from the origin (0.5–25 m) were selected. The point cloud of these 50,000 planes and 50,000 lines containing random instrumental measurement errors were simulated.

Point clouds were simulated by projecting rays in angular increments according to the angular resolution of the TLS instrument used in this study. These rays are then intersected with each simulated plane (or line) to identify the range of the noise-free observations. Random measurement errors were incorporated for both angular and range measurements. The random measurement errors were assumed to be normally distributed, $N\left(0,\sigma^{2}\right)$, with measurement precision (σ) set according to the manufacturer’s specifications. The Cartesian coordinates of each point is then calculated. Next, the attributes, defined in Section 3.2.1, were estimated with the 35 mm spherical neighbourhood. The percentage of relative error for each of the identified attributes from the error-free attributes was calculated.

Figure 3a,b shows the 95th percentile (approximately 2*σ*) of the percentage of relative error for each plane/line attribute versus the distance of the plane/line to the origin. The directional vector components ($\vec{d}$; Figure 2) of the simulated linear points are affected less by the point measurement errors compared to the normal vector of the simulated planar points. In the work of [61], it was shown that, in cases where the largest eigenvalue is larger than the error variance (such as the case of a linear feature), the relative impact of the measurement error on the eigenvector corresponding to the largest eigenvalue is small, which agrees with the results presented here. The best fit line for each of the curves was used to determine the value of the initial threshold.

The similarity thresholds in Figure 3 are small enough to prevent under-segmentation as they only incorporate the impact of random measurement errors. Therefore, this process may result in over-segmentation. To minimize over-segmentation and help reduce the dependency of the segmentation on the choice of the initial threshold, the “*robust complete linkage*” is developed to merge the over-segmented groups of points.

#### 3.2.4. Robust Complete Linkage

First, the clusters with the closest attributes are identified, say clusters *U* and *V* with sizes $N_{U}\leq N_{V}$ (number of points within each cluster). A subset of observations from cluster *U* (no more than 25% of $N_{V}$) with the smallest Mahalanobis distance [63] in cluster *U* are added to cluster *V*. 25% outlier mark is used since it provides a good balance between efficiency and breakdown value [21]. Det-MCD is applied to the newly formed cluster, and the robust covariance matrix (${\overset{\text{\textasciicircum{}}}{\Sigma }}_{DetMCD}$) and centre (${\hat{\mu }}_{DetMCD}$) are estimated. The outliers of the newly merged cluster are determined as follows:

(5) $${\mathrm{Mah}}^{2}\left(X_{i}\right)=\left(X_{i}-{\hat{\mu }}_{DetMCD}\right){{\overset{\text{\textasciicircum{}}}{\Sigma }}_{DetMCD}}^{-1}{\left(X_{i}-{\hat{\mu }}_{DetMCD}\right)}^{T}:\{\begin{array}{cc} \begin{array}{c} <\chi_{3,0.975}^{2}\\ >\chi_{3,0.975}^{2} \end{array} & \begin{array}{c} \mathrm{Inlier}\\ \mathrm{Outlier} \end{array} \end{array},$$

where ${\mathrm{Mah}}^{2}\left(X_{i}\right)$ is the squared 3D Mahalanobis distance of observation i=1:n, $X_{i}$ is the vector of Cartesian coordinates of observation i, and $\chi_{3,0.975}^{2}$ is the chi-squared value with 3 degrees of freedom (3-dimensional data) and confidence of 97.5%. Equation (5) suggests that any observation whose Mahalanobis distance is larger than $\chi_{3,0.975}^{2}$ (which is a well-established convention used in robust statistical approaches [21]) is considered an outlier.

**Figure 5:**
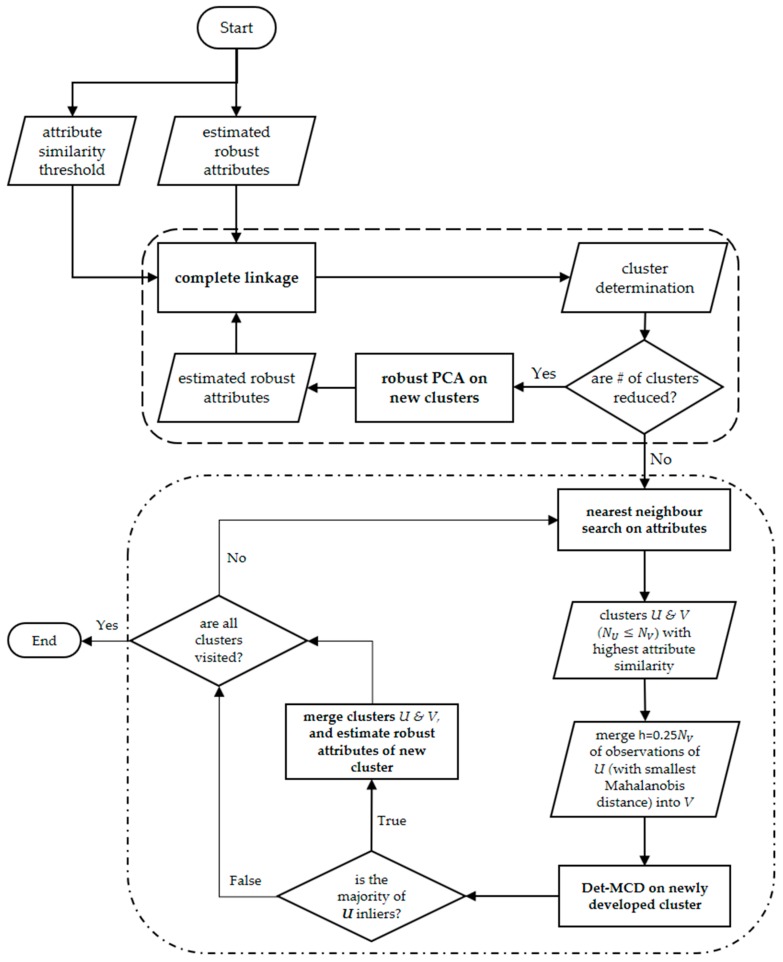
Robust complete linkage algorithm for robust clustering of planar and linear surfaces.

The two clusters *U* and *V* are merged if and only if more than 50% of the points from cluster *U* are identified as inliers. In other words, the cluster is rejected when the majority of the outliers are from that cluster. The aforementioned steps are then applied to the merged cluster *UV* until no other cluster can be merged. The robust complete linkage procedure is then repeated for the remaining clusters. The algorithm continues until every cluster is visited at least once. To improve the computational efficiency, clusters with attribute similarity larger than 20% of the attributes of the base cluster (cluster containing more points) are not examined. Figure 4b provides a schematic representation of the “*robust complete linkage*” algorithm in 2D. Figure 5 shows the flowchart of the proposed “*iterative complete linkage*” and “*robust complete linkage*” algorithms together.

#### 3.2.5. Spatial Continuity of Clusters

Using the proposed segmentation method, it is possible to group together spatially discontinuous surfaces since spatial connection has not yet been considered. To enforce surface continuity and connectivity of clusters, the inner (openings/windows) and outer boundary points for each linear and planar segment are determined using α-shapes [64,65]. First, the points in each cluster are projected onto the best-fit surface to reduce the dimensionality of the data. For each projected surface, the critical radius, the smallest radius through which the α-shape encloses all points, is calculated. Using this critical radius, the neighbouring α-extreme points are determined and joined together to define the inner and outer boundaries. Points between the inside and outside boundaries are considered as one segment.

### 3.3. Robust Extraction of Flat Slab Floors

Flat slab floors and columns are common structural elements on building construction sites. Here, a new method is proposed to identify and extract points of flat slab floors and ceilings using the histogram of point height prior to the application of the proposed robust PCA. This is particularly beneficial to reduce the number of points to be segmented and consequently helps decrease the calculation time of the proposed segmentation procedure. A similar idea was also introduced in [66] to extract the interior floors and ceilings of a completed building using the histogram of point height. Here, a robust floor and ceiling extraction method using the histogram of point height is proposed, which minimizes the dependency of the algorithm on a specific thresholds value.

**Figure 6:**
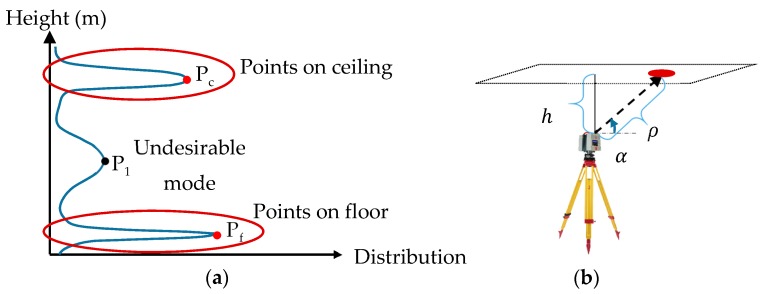
(**a**) Expected point height distribution; (**b**) schematic representation of the raw measurements of a point on a horizontal plane.

A typical histogram of point height for a room or a construction site with flat slab ceiling and floor is schematically shown in Figure 6a. The schematic histogram comprises two major peaks, $P_{f}$ and $P_{c}$ as well as a minor peak, $P_{1}$. $P_{f}$ and $P_{c}$ are modes representing points on the floor and ceiling respectively. Point $P_{1}$ most likely represents a region of high point density or a smooth, non-planar surface. The objective is to distinguish the two major peaks, $P_{f}$ and $P_{c}$, from other undesirable modes. The first step is to locate the peaks using a mode-detection algorithm. Here, the median-shift method proposed in [67] was adopted since no a priori knowledge of the number of modes is required and it is robust to outliers within the dataset. Median-shift will detect all three peaks shown in Figure 6a. Hence, to distinguish modes $P_{f}$ and $P_{c}$ from mode $P_{1}$, a cut-off value on the robust standard deviation of the vicinity of the identified modes must be determined.

The value of this threshold is defined with respect to the measurement errors of points on flat slab ceiling (or floor) using the law of variance propagation and the TLS instrument specifications. Figure 6b shows a schematic representation of a ceiling (or floor) relative to a levelled TLS instrument. Using the notations presented in Figure 6b, the height precision can be expressed as a function of range and vertical angle precision, assuming no correlation between the range and vertical angle errors (scanner is assumed pre-calibrated):

(6) h=ρ.sinα,

(7) $$\sigma_{h}^{2}={\left(\frac{\partial h}{\partial \rho }.\sigma_{\rho }\right)}^{2}+{\left(\frac{\partial h}{\partial \alpha }.\sigma_{\alpha }\right)}^{2},$$

(8) $$\sigma_{h}^{2}=\sin^{2}\alpha .\sigma_{\rho }^{2}+\rho^{2}.\cos\alpha^{2}.\sigma_{\alpha }^{2},$$

(9) $$\rho =\frac{h}{\sin\alpha }\to \;\sigma_{h}^{2}=\sin^{2}\alpha .\sigma_{\rho }^{2}+h^{2}.\cot^{2}\alpha .\sigma_{\alpha }^{2},$$

where h, ρ and α are the height, range and angle of the laser beam of the point to the horizontal plane, respectively; $\sigma_{\rho }$ and $\sigma_{\alpha }$ are the standard deviations of the range and horizontal angle, respectively; and $\sigma_{h}$ is the standard deviation of the derived height. The range precision ($\sigma_{\rho }$) is a function of range and incidence angle. The impact of incidence angle along with range on range precision can be modelled as a function of the range precision at normal incidence following the formulation provided by ([68]; Equation (17)):

(10) $$\sigma_{\rho }=\frac{\sigma_{\rho_{⊥}}}{\sin\alpha }.\frac{\rho_{max}^{2}-\rho_{min}^{2}}{\rho_{max}^{2}-\rho^{2}},$$

where $\rho_{max}$ is the maximum unambiguous range of the scanner, $\rho_{min}$ is the minimum range to receive backscattering signal, and $\sigma_{\rho ⊥}$ is the range precision at normal incidence at $\rho_{min}$ from the scanner. By substituting Equations (6) and (10) into Equation (9), the following equation is formulated:

(11) $$\sigma_{h}^{2}={\left(\frac{\rho_{max}^{2}-\rho_{min}^{2}}{\rho_{max}^{2}-{\left(\frac{h}{\sin\alpha }\right)}^{2}}\right)}^{2}.\sigma_{\rho_{⊥}}^{2}+h^{2}.\cot^{2}\alpha .\sigma_{\alpha }^{2},$$

The coplanar points of the ceiling (or floor) comprise the locus of points in space with the same height from the origin; therefore, h, the height of the identified mode estimated using the median-shift algorithm, in Equation (11) is constant since the scanner is assumed to be level. Furthermore, $\sigma_{\rho ⊥}$, $\rho_{max}$, and $\rho_{min}$ are dependent upon the scanner used for data collection and are typically reported by the manufacturer. As a point of reference, the value of $\sigma_{\rho_{⊥}}.\frac{\rho_{max}^{2}-\rho_{min}^{2}}{\rho_{max}^{2}-\rho^{2}}$ for the TLS used in this study, Leica HDS6100, at 25 m and 50 m from the scanner is less than 1 mm and 2 mm, respectively [69]. Therefore, $\sigma_{h}$ can be expressed as only a function of α as in Equation (11). As observed, $\sigma_{h}$ increases as the angle α increases (or decreases) from $\frac{\pi }{2}$ to π (or zero). Consequentially, by considering Equation (5), it can also be concluded that $\sigma_{h}$ increases as ρ increases. Therefore, the smallest (or largest) angle for α in the vicinity of an identified mode dictates the maximum value for $\sigma_{h}$. A trial and error process is adopted to identify the expected smallest (or largest) value of α for each of the identified modes, and subsequently determine the $\sigma_{h/max}$.

The $\sigma_{h/max}$ obtained from the aforementioned process is used to remove the undesirable modes. The robust standard deviation for the univariate height of the identified neighbouring points is calculated using the normalized median absolute deviation (MADN; [70]):

(12) $$MADN=\frac{\mathrm{Median}\left(\left|\vec{H}-\mathrm{Median}\left(\vec{H}\right)\right|\right)}{0.67449},$$

where $\vec{H}$ is the univariate height data represented as a column vector. To distinguish major peaks from the insignificant mode, the value of the MADN must be smaller than $\sigma_{h/max}$. This condition eliminates groups of points such as those in the vicinity of $P_{1}$.

After the significant modes are detected, points within $\pm 3\sigma_{h/max}$ [71,72] from the modes are chosen. To further refine the results and choose only the coplanar points with the same height, for each observation, the 1D Mahalanobis distance is calculated as follows:

(13) $${\mathrm{Mah}}_{i}^{2}={\left(\frac{H_{i}-h}{MADN}\right)}^{2},$$

where h is the value of the defined mode of the height and $H_{i}$ is the height of observation i. The observations whose Mahalanobis distance is smaller than $\chi_{1,0.975}^{2}$ are considered the final set of observations. This final step reduces the occurrence of Type II errors in extracting points belonging to the floors or ceilings [43]. After these points are detected for each scan separately, the robust PCA is performed on the remainder of registered points to complete the planar and linear classification processes. Each extracted floor segment is then treated as one cluster within the robust complete linkage method.

### 3.4. Method of Validation of Results

To assess the quality of the segmentation, and classification, the precision, recall and accuracy are estimated using the convention presented in [73]:

(14) $$Precision=\frac{T_{P}}{T_{P}+F_{P}},$$

(15) $$Recall=\frac{T_{P}}{T_{P}+F_{N}},$$

(16) $$Accuracy=\frac{T_{P}+T_{N}}{T_{P}+T_{N}+F_{P}+F_{N}},$$

where $T_{P}$, $T_{N}$, $F_{P}$, and $F_{N}$ are the number of true positive, true negative, false positive and false negative counts. For the classification results, Equations (14)–(16) were used to report the precision, recall and accuracy for two classes of features, i.e., planar and linear (the remaining points were marked as neither planar nor linear, and not utilised during the segmentation process). For reporting segmentation quality, the precision, recall and accuracy values were calculated considering only the points classified as planar (or linear). The ground truth values of the planar and linear features, and segments were extracted manually. To evaluate the effectiveness of the proposed robust complete linkage, similar to [8,32], the linear and planar segmentation results using our method were compared to the results of the region growing method proposed by Rabbani [47]. Rabbani’s region growing method was used here for comparison since it is one of the most cited and accepted region growing methods.

## 4. Experiment Description

Three sets of experiments were designed to demonstrate the effectiveness of the proposed robust planar and linear segmentation. The results from eight datasets collected using a Leica HDS6100 TLS are presented. The first experiment was the as-built modelling of the Mechanics of Materials Laboratory (MML) at the University of Calgary to assess the applicability of the proposed algorithms in a small but complex environment before field data on construction sites were collected. The second experiment was construction progress monitoring of the Graduate Student Hall of Residence (GSHR) building, which mainly consists of reinforced concrete structural elements with flat slab floors, and rectangular cross-section columns. The third experiment was the dimensional compliance control for the Taylor Institute of Teaching and Learning (TITL) building, which features a unique skeletal steel structure composed of rectangular/square Hollow Structural Sections (HSS).

To register TLS point clouds acquired at different points in time, signalized targets over pre-surveyed control points were used. At least four non-collinear targets were within the line-of-sight (LOS) of every instrument location. The registration precision, estimated through the Mean Radial Spherical Error (MRSE), for the eight datasets presented in this manuscript is provided in Table 1.

The methods proposed in this manuscript were implemented in Matlab programming language using the Parallel Processing Toolbox, and analysed through a computer with Intel Core i7-4930K @ 3.40 GHz CPU, 32 GB of DDR4 RAM, 500 GB of SSD, and 2 GB of dedicated graphics card.

### 4.1. Experiment 1: Mechanics of Material Laboratory

**Figure 7:**
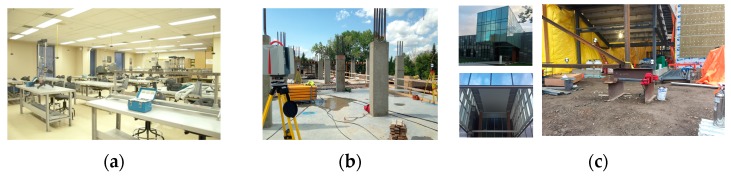
(**a**) MML laboratory; (**b**) GSHR construction site; and (**c**) TITL building.

One composite point cloud comprising the data captured from three scan stations was collected from the MML (Figure 7a). As illustrated, the laboratory consists of many obstacles including metallic tables that cause occlusions and multipath reflection. Therefore, it provided a fair representation of an indoor construction site, especially to assess the applicability of the proposed segmentation methods before field data were acquired.

### 4.2. Experiment 2: Graduate Student Hall of Residence Construction Site

The GSHR construction site shown in Figure 7b was monitored for a duration of six weeks. Six TLS datasets were collected, roughly one every week, to record the progress of construction activities on a specific portion of the site. These six datasets comprised data from the first four floors of the building. The main structure of the building consists of concrete columns with rectangular cross-sections (planar façades), flat slab floors/ceilings, and reinforcement bars (rebar). Therefore, planar surfaces (flat slab floors, ceilings, and column façades) and linear features (rebars) had to be segmented. Preliminary examination of the point clouds revealed the presence of many outlier points that had to be automatically identified and removed from the points of the main structure. The presence of safety guardrails, shoring and other objects on site also occluded the LOS of the scanner to some elements of the site, resulting in missing data. The applicability of the methods proposed in Section 3 for robust segmentation of the planar and linear features of the contaminated TLS datasets was examined.

### 4.3. Experiment 3: Taylor Institute of Teaching and Learning Construction Site

The third experiment was the monitoring of the TITL construction site (Figure 7c). The objective was to model the main truss elements and control the compliance of their dimensions relative to the specifications of the project plan. This unique building consists of a large skeletal steel structure truss, and many other steel elements with planar facets such as stairs and H-section elements. The main truss consists of steel elements with planar-rectangular facets (hollow square cross-section). One TLS dataset comprising six scan stations is presented from this site. The point cloud contained many outlier points and, thus, provided a suitable dataset for the validation of our proposed planar segmentation method.

## 5. Experimental Results

### 5.1. Experiment 1: Mechanics of Materials Laboratory

**Figure 8:**
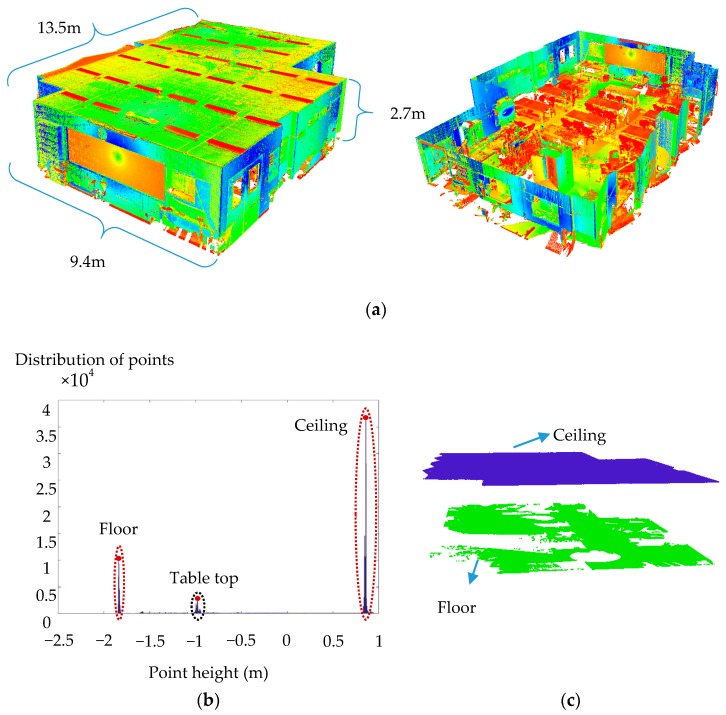
(**a**) Registered point clouds acquired from the MML (left, outer view; right, sliced view of the inside); (**b**) histogram of point height of the point cloud acquired from the MML; and (**c**) results of the automatic floor and ceiling extraction (each colour represents a different segment).

Figure 8a shows the registered points of the acquired point cloud, consisting of three scan-stations with over 30 million points with colour-coded return signal intensity. The points of the floor and the ceiling were first extracted using the method proposed in Section 3.3. The planar surfaces of the remaining points were then classified and segmented using the robust PCA classification and robust iterative complete linkage segmentation methods. The results of each step are provided in the following subsections.

#### 5.1.1. Robust Floor and Ceiling Extraction

The histogram of point height is shown in Figure 8b, which complies with the hypothesized distribution in Figure 6a. The identified modes of the histogram of point height using the median-shift algorithm are shown with red dots in Figure 8b. As illustrated, three modes were detected, representing the floor, ceiling and points on the metallic tables. Figure 8c shows the final results of the identified points on the floor and ceilings. Using the proposed criteria, it was possible to correctly differentiate between the points on the floor/ceiling from those of the tables, which carried larger *MADN* in the vicinity of the detected mode.

Approximately 18 million points were identified and extracted as belonging to the floor and the ceiling, which accounted for more than half of the total points. The computation time of the robust PCA classification is nearly four times that of the proposed floor extraction method for the same number of points. This is attributed to the higher computational complexity of the nearest neighbour search in 3D as well as the Det-MCD estimation of 3D data during the robust PCA classification compared to the 1D nearest neighbour search and the univariate median-shift algorithm for the proposed floor extraction. Therefore, the use of this method can help improve the computational complexity of the proposed planar classification algorithm. The precision, recall and accuracy of the extracted points were 95.2%, 100.0% and 96.4% for the floor and 94.6%, 100.0% and 95.3% for the ceiling, respectively. These outcomes demonstrate the robustness of the proposed method, especially to Type II errors. Therefore, the proposed method reduces the computational time while providing robust floor extraction results.

#### 5.1.2. Robust Planar Classification and Segmentation

**Figure 9:**
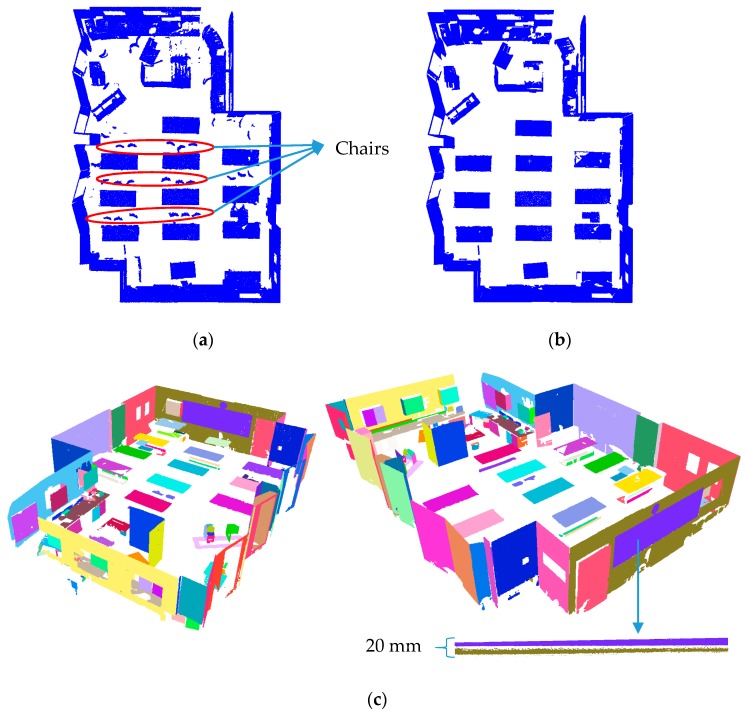
Planar classification of MML point cloud: (**a**) using only predefined thresholds of Equation (3); and (**b**) using outlier detection (Det-MCD) on the normalized eigenvalues of classified points of (**a**). (**c**) Robust planar segmentation of the classified planar points of (**b**).

Figure 9a shows the results of the planar classification using the thresholds given in Equation (3). The precision, recall and accuracy of the classified planar surfaces at this stage were 96.5%, 90.8% and 88.2%, respectively. The recall rate reflects the fact that some points of non-planar surfaces, such as chairs, were misclassified as planar (approximately one million points). Figure 9b shows the impact of the refinement stage presented in Section 3.1.2, which is the application of the Det-MCD on the eigenvalues of the classified points of Figure 9a to identified the outliers of the eigenvalues. As can be seen, many of the incorrectly-classified points were effectively removed to reduce the Type II errors. The precision, recall and accuracy after the refinement stage were 94.3%, 98.1% and 93.4%, respectively.

The reduction of Type II errors in the planar classification also improves computational efficiency since fewer non-planar points are considered during the segmentation process. The robust complete linkage was applied on the classified planar points shown in Figure 9b to determine coplanar points. Figure 9c shows the result of the planar segmentation. Each colour represents a different segment. The magnified purple and green segments represent the blackboard and the wall of separated by only 20 mm. This provides an indication of the ability of the proposed segmentation algorithm to distinguish between two globally proximate planes separated by a small distance. The precision, recall, and accuracy of the planar segmentation were 95.8%, 98.2%, and 94.2%, respectively.

### 5.2. Experiment 2: Graduate Student Hall of Residence

**Figure 10:**
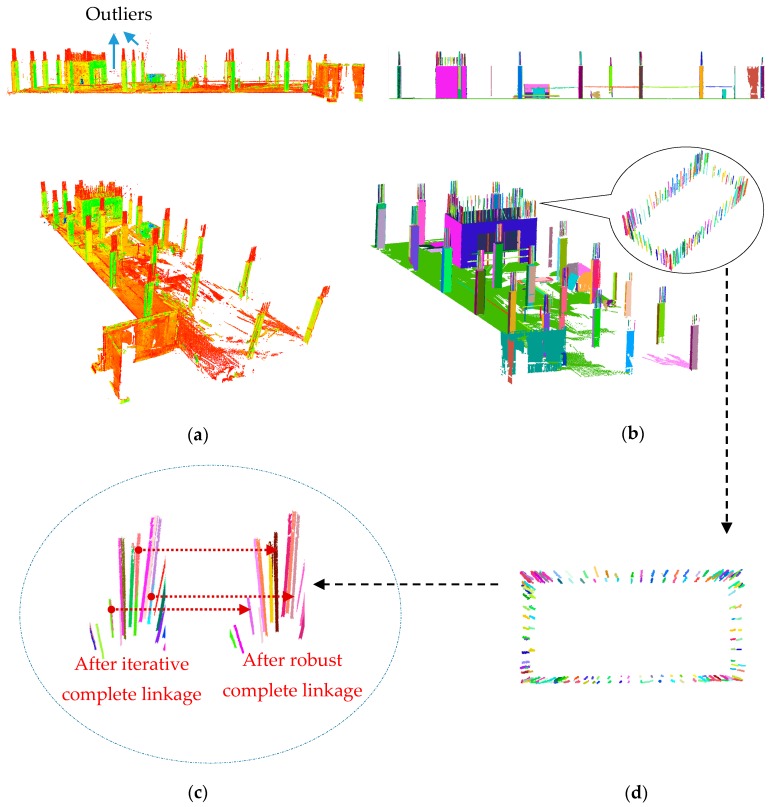
(**a**) Point cloud captured from Epoch 1 (colour represents intensity); (**b**) robust planar and linear segmentation results (each colour represents one segment); (**c**) rebar segmentation before and after the robust complete linkage algorithm; and (**d**) top-view of the rebar (linear feature) segmentation of the elevator shaft.

Figure 10a illustrates the registered point cloud of the first floor of the GSHR building captured during the first site visit (Epoch 1). Figure 10b shows the planar and linear segmentation results of for Epoch 1 following the application of the methods presented in Section 3.

#### 5.2.1. Epoch 1: Linear Segmentation Results

As indicated in Section 3.2, first the iterative complete linkage was carried out, followed by the robust complete linkage to reduce over-segmentation. To illustrate the importance of eliminating the choice of the similarity threshold, linear segmentation using the iterative complete linkage is compared to that after the implementation of the robust linkage. Figure 10c shows the results of the merging before and after the robust complete linkage. It can be seen that, among the 10 rebars shown, the iterative complete linkage resulted in 14 segments, whereas the robust complete linkage identified exactly 10 segments—no over-segmentation. Overall, from 357 rebars, the iterative complete linkage provided 498 segments and robust complete linkage provided 362 segments.

The ability of the robust complete linkage algorithm to cluster planar segments was briefly discussed in our previous work [43], where over 95% of the planar points were segmented correctly. For Epoch 1, similar planar segmentation results were achieved and the precision, recall and accuracy of the planar segmentation were 97.6%, 98.1%, and 96%, respectively. In [43], a linear segmentation method was proposed that used two different origins to define the attributes of a linearly classified point. This method was able to correctly segment just under 87% of the linearly identified points. However, the results may be affected by the arbitrarily-selected location for the second origin. The more robust approach for estimating the attributes of linearly classified points introduced (Section 3.2) does not require the selection of a secondary origin. Using this new method for attribute definition, the linear segmentation precision was improved to 95.7%.

#### 5.2.2. Epoch 1: Comparative Evaluation of Rebar Segmentation Using Robust Complete Linkage

Figure 10d shows the top view of the segmented linear features (rebars) of the elevator shaft. As can be seen, the rebars are extremely close to each other (about 25 to 30 mm apart). Examination of the directional vectors of the linearly classified points revealed that the subtended angle between vectors of more than 96% of the neighbouring points was less than 1.5°. In this situation, region growing alone does not yield to reasonable results since the choice of neighbourhood size and the choice of angular threshold used to merge neighbouring points with similar attributes is important. A larger similarity threshold and a larger neighbourhood size may result in under-segmentation, both of which are usually subjectively defined and may not be generalizable for other datasets.

**Figure 11:**
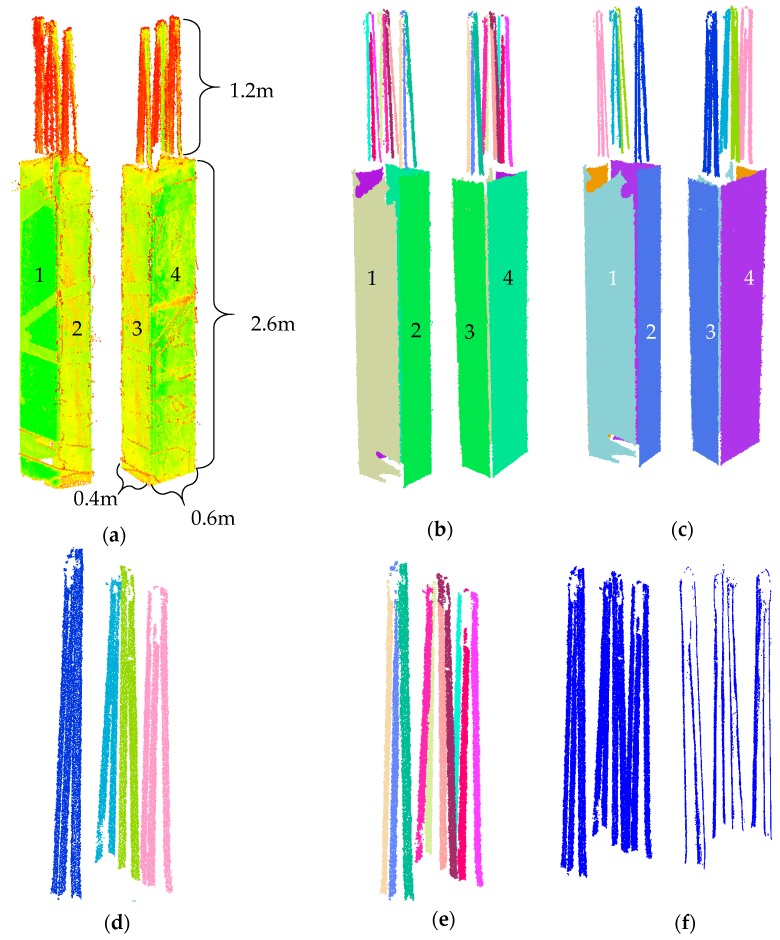
(**a**) Point cloud of one of the columns; (**b**) planar and linear segmentation results using our method; and (**c**) planar and linear segmentation results using region growing of [47] with robust PCA. Linear segmentation results of the rebars using: (**d**) Rabbani et al. region growing with robust PCA classification; and (**e**) our method. (**f**) Classified linear points: (left) Cartesian coordinates; and (right) coordinates of the robust centres.

To further demonstrate, the point cloud of one of the columns from Epoch 1 was selected (Figure 11a), and the result of segmentation using robust complete linkage was compared to that of Rabbani et al. [47]. We use the results of the robust PCA linear and planar classification (Section 3.1) in both cases to provide a fair segmentation comparison. For Rabbani’s method, the threshold for the angle created between the normal or directional vectors of adjacent points (smoothness constraint) was initially set to 10°. Considering the planned placement of the rebars (30–35 mm apart) and planar surfaces, the region growing neighbourhood size was set to 25 mm or 30 closest neighbours, whichever is smaller.

The segmentation results using our method and the region growing method of Rabbani are presented in Figure 11b,c, respectively. The planar segmentation results did not vary significantly since the angle between the normal vectors of two adjacent planes were much more than 10° (close to 90°), and the distance of two parallel planes were more than 25 mm (approximately 400 mm) apart. The linear segmentation results however were significantly different. As illustrated in Figure 11d,e, only four segments were identified using Rabbani’s method compared to the 10 segments correctly identified by our method. This shows the under-segmentation of the linear features using the implemented region growing method. It is important to mention that the linear segmentation result using Rabbani’s method provided identical results when the smoothness threshold was reduced to 5° and neighbourhood size reduce to 20 mm. In this example, the computation time for ours and Rabbani’s methods were 1632, and 1143 s respectively.

As indicated in Section 3.2.1, the robust centres of the neighbourhood of each point were utilized to define the planar and linear attributes. Figure 11f (left) shows the arrangement of the Cartesian coordinates of points classified as linear for the subset presented in Figure 11a. Figure 11f (right) shows the robust centres of the same points. Using robust centres allows for the determination of the axis of the cylindrical rebars, which follow a linear pattern. Hence, the robust centres provide a more efficient means to differentiate between different linear features (Section 3.2.1).

#### 5.2.3. Epochs 1–6: Robust Floor Extraction

**Figure 12:**
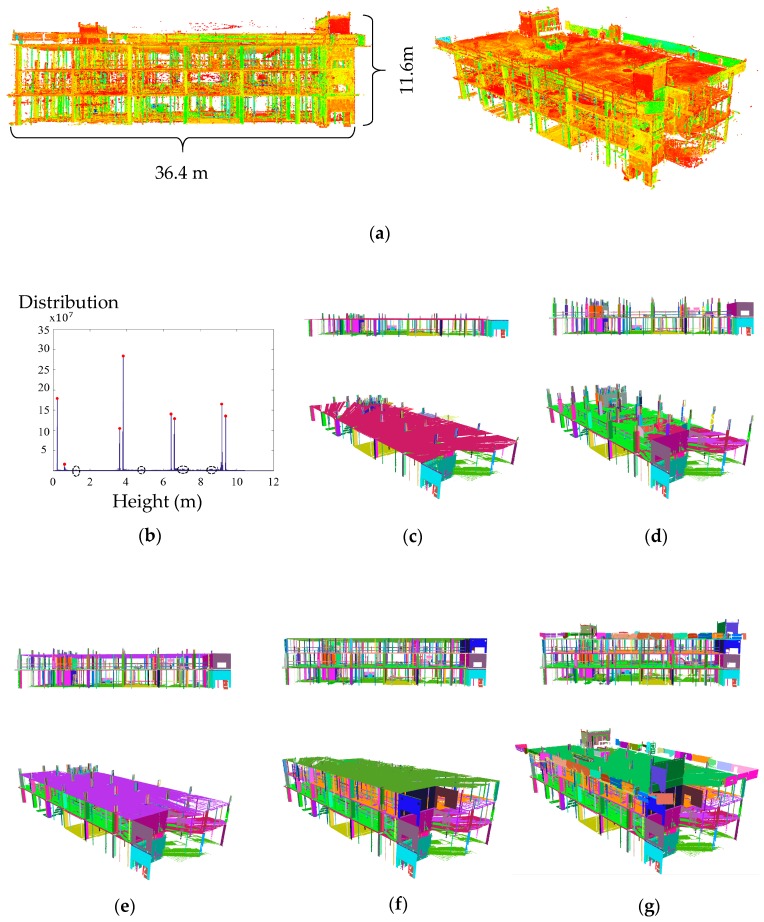
(**a**) The point clouds of the first four floors (all six epochs) registered to the reference coordinate system; and (**b**) histogram of point height of all of the scans combined; planar and linear segmentation results (top and side views) from the accumulated points of: (**c**) Epochs 1–2; (**d**) Epochs 1–3; (**e**) Epochs 1–4; (**f**) Epochs 1–5; and (**g**) Epochs 1–6.

Figure 12a shows the registered point clouds of all six TLS datasets collected from the site (i.e., Epochs 1–6). Figure 12b shows the corresponding distribution of point height of the data presented in Figure 12a. Using the median-shift mode detection algorithm, 12 peaks were identified, which are shown as red dots and dashed ovals in Figure 12b. The proposed method was able to distinguish all significant peaks, (from the insignificant/undesirable ones. More than 1.7 billion points were identified as floor and ceiling points. The precision, recall and accuracy of the extracted points on flat slab floors and ceilings were 94.7%, 100.0% and 97.2%, respectively.

The 100.0% recall rate for all datasets demonstrates that the method is robust to Type II errors. After the flat slab point extraction, the remaining points (including the 5.3% of points on the flat slab ceiling and floors that were not identified as points on floor or ceiling) were classified into planes and lines, and then merged together using the proposed robust segmentation algorithm. Among the points that were not correctly identified as points on floors or ceilings within our initial flat slab identification method (Type I errors), approximately 38.3% were correctly merged to the initially extracted points using the proposed robust complete linkage algorithm.

#### 5.2.4. Epochs 2–6: Robust Planar and Linear Segmentation

Figure 12c–g shows the results of the planar and linear segmentation of the complete construction site. The summary of the classification and segmentation results for each epoch is given in Table 2. As shown, consistent planar and linear classification and segmentation results were achieved for all six sets of data. The segmentation results were based on the planar and linearly classified points. An overall accuracy of 96.1% was achieved for both planar and linear segmentation.

### 5.3. Experiment 3: Taylor Institute of Teaching and Learning

**Figure 13:**
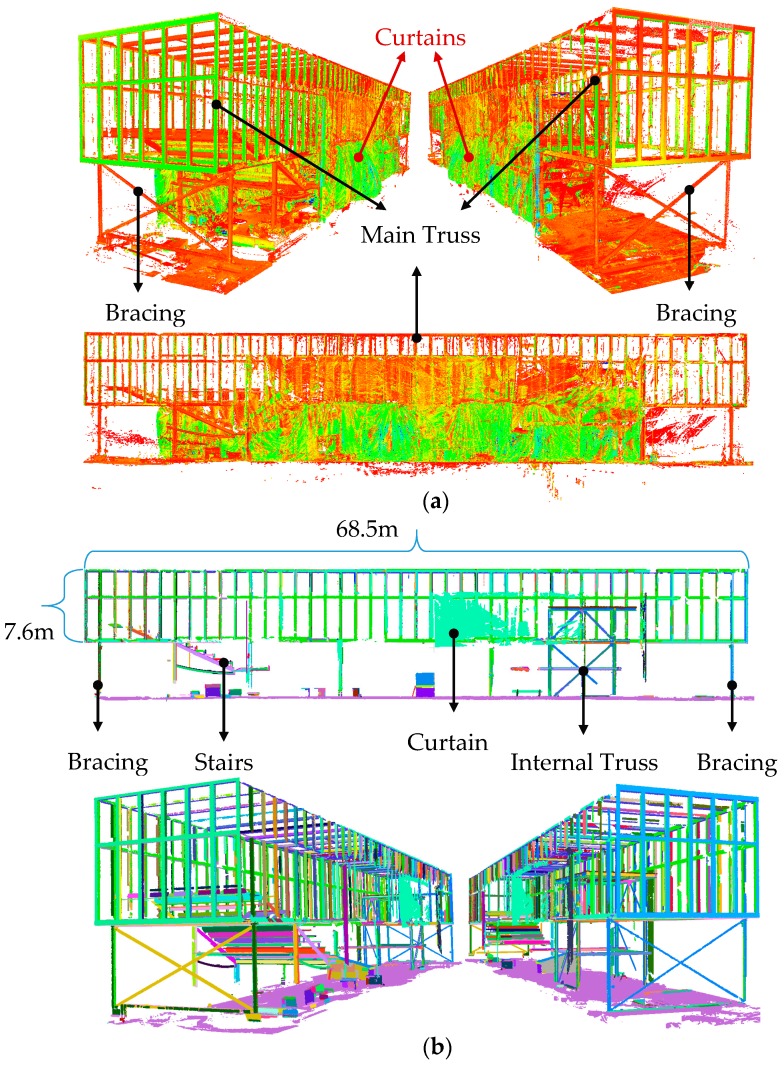
(**a**) point cloud of the TITL building during construction; and (**b**) result of the robust planar segmentation of the whole site.

The registered point cloud acquired from the TITL construction site, consisting of six scan stations with overall registration precision of 1.7 mm (Table 1), is shown in Figure 13a. The point cloud contains temporary bracings. Curtains were also attached to each side of the main truss to help retain heat within the construction site Therefore, the applicability of the proposed robust planar classification and segmentation in identifying the randomly shaped curtains along with the many outliers were thoroughly investigated.

Figure 13b shows the robust planar segmentation results of the overall site consisting of stairs, beams, bracings, the internal truss and the main truss. The main truss consists of steel elements with hollow square cross-sections (square tube). The precision, recall and accuracy of the robust planar classification were 93.4%, 92.4%, and 93%, respectively. The segmentation precision, recall and accuracy of the classified planar points of the main truss were 95.4%, 96.3%, and 92.5%, respectively. The robust planar segmentation results of each of the remaining components are presented in more detail in the following subsections.

#### 5.3.1. Stairs: Robust Planar Segmentation

**Figure 14:**
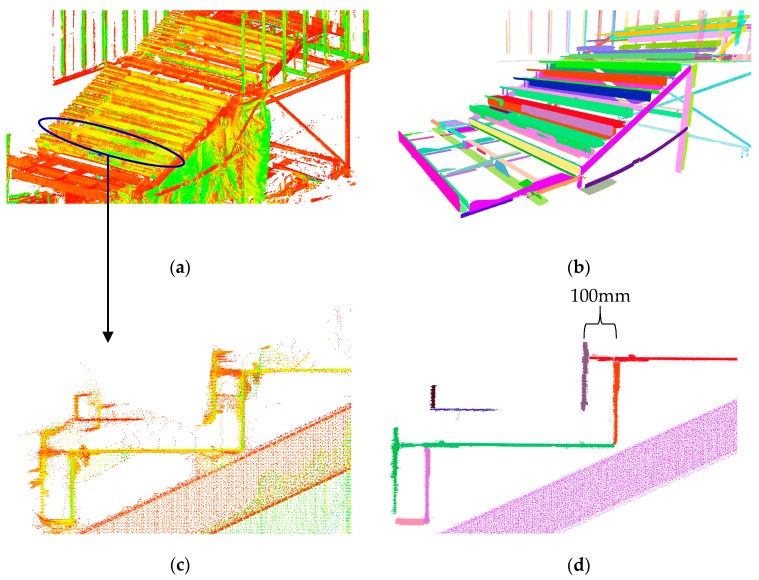
(**a**) Point cloud of the staircase; (**b**) robust planar segmentation results of the staircase; (**c**) point cloud of the random subset of (**a**); and (**d**) planar segmentation results of the point cloud of (**c**).

As shown in Figure 13b, the TITL building consists of a staircase with H-sections elements as the main structure, and welded planar plates as the elements of the stairs. Figure 14a shows the point cloud of the whole staircase and Figure 14b shows the results of the planar segmentation. Figure 14c shows the magnified version of a subset of the point cloud presented in Figure 14a. As illustrated, the point cloud consists of many outlying points. Figure 14d shows the robust planar segmentation results of the point cloud presented in Figure 14c. The proposed robust classification and segmentation could eliminate many of the outlying points (approximately 95.2% in the case of the stairs). Our proposed robust planar segmentation was also capable of easily differentiating between the front and back plates of the staircase of Figure 14c, which are 100 mm apart. The precision, recall, and accuracy of the segmentation of the planar points of the staircase were 94.7%, 94.3%, and 90.1%, respectively.

#### 5.3.2. Internal Truss: Robust Planar Segmentation

**Figure 15:**
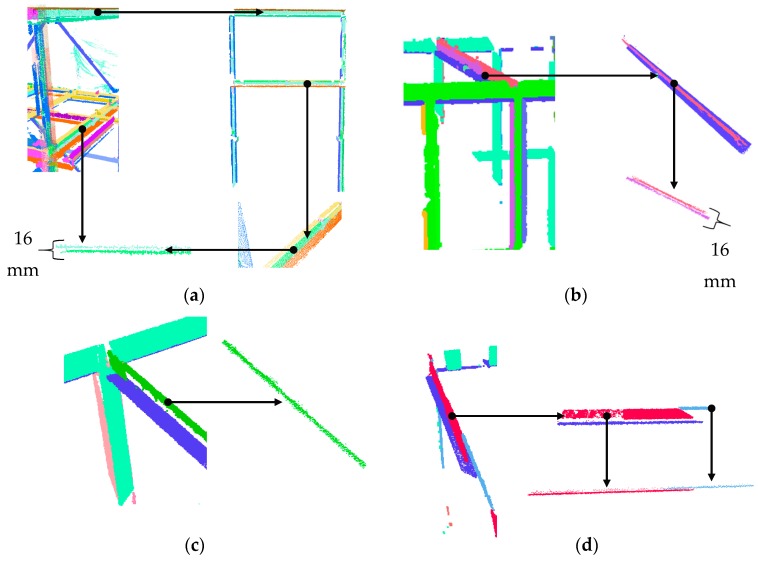
(**a**) Results of the segmentation of the internal truss frame consisting of H-section elements. Results of the robust planar segmentation of the H-section beams of the top of the main truss: (**b**) case where the two surfaces were correctly identified; (**c**) case where the high level of noise did not allow for correct differentiation of the surfaces; and (**d**) case where the density of the accumulated points on one surface did not allow for the correct segmentation of the two faces of the planar web.

The internal trusses, shown in Figure 13b, consist of H-section elements. The thickness of the plates constituting the web of the H-section elements are 16 mm. Here, the effectiveness of the robust planar segmentation method in identifying both sides of the plates is examined. Figure 15 provides a summary of these results. Figure 15a shows a frame that consists of four H-section elements. It is possible to differentiate between the two planar faces of a single web using the proposed robust complete linkage segmentation method. In this particular H-section, approximately 88.7% of the points on the web were segmented correctly.

Figure 15b shows a sample of an H-section beam located on the top of the main truss. As can be illustrated, the two surfaces are clearly distinguished in this case, and more than 90.2% of the points were segmented correctly. Figure 15c demonstrates an H-section beam where the large noise in the measurement has not allowed for the robust segmentation to distinguish between the two planar faces of the web. Figure 15d shows an H-section beam where the blue surface is visually distinguishable from the red surface. However, these points were incorrectly assigned to the red surface due to the very low point density. Overall, amongst the 53 H-section elements, the webs of 47 were observed from both sides. From the 94 (47 × 2) potential web planar surfaces, 80 of them were correctly identified. From all the points of the 53 H-section elements, approximately 87.1% were correctly segmented. The precision, recall and accuracy of the planar points belonging to the small truss were 97%, 92.3%, and 91.2%, respectively.

#### 5.3.3. Evaluation of H-Section Web Segmentation Using Robust Complete Linkage

**Figure 16:**
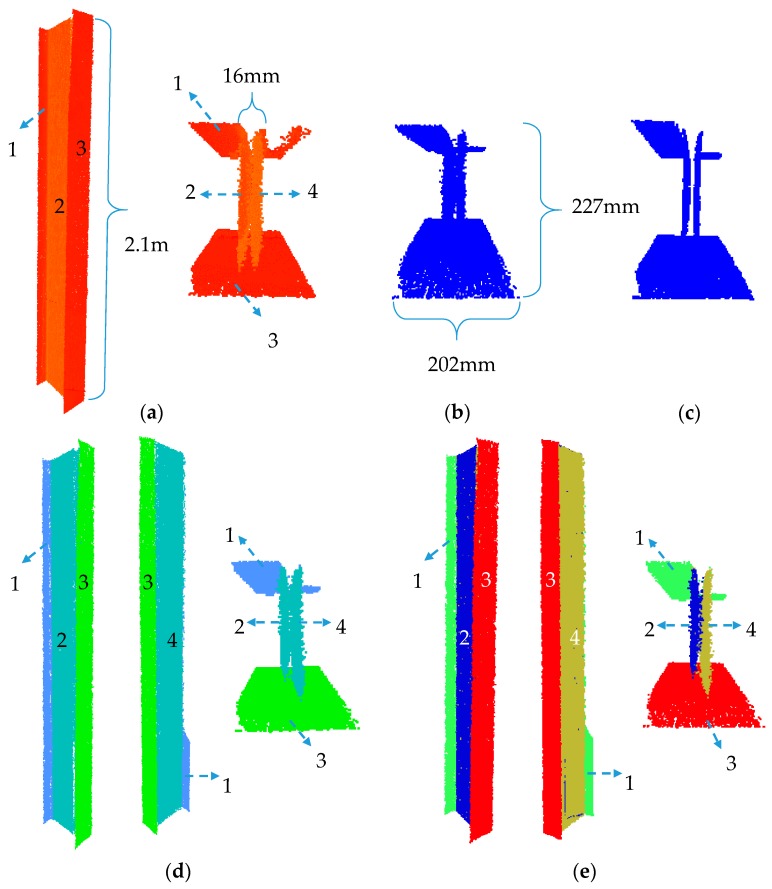
(**a**) Point cloud of a sample H-section column; (**b**) points classified as planar using robust PCA; (**c**) the robust centres of the planar classified points; (**d**) planar segmentation results using [47] method; and (**e**) planar segmentation results using our proposed method.

To show the effectiveness of our proposed method in distinguishing the planes of the web of H-section elements, the point cloud of a subset of an H-section column is used (Figure 16a). The planar features were segmented using both our method and Rabbani’s region growing method. Similar to Section 5.2.2, the results of the robust PCA were used for both cases to make a fair comparison of the segmentation results. Figure 16b shows the points that were classified as planar using the robust PCA. Figure 16c shows the robust centres of the neighbourhood of the same points. As illustrated, the robust centres allow differentiation between the two web planes, which demonstrates the effectiveness of using robust centres as opposed to the Cartesian point coordinates.

The angular threshold and neighbourhood sizes for the region growing were set to 10° and 10 mm (or 30 closest points, whichever is smaller), respectively. The neighbourhood size was purposely chosen to be smaller than the thickness of the web to minimize over-segmentation. The results of the planar segmentation using our method and Rabbani’s region growing method are presented in Figure 16d,e. As illustrated, Rabbani’s region growing method was not capable of distinguishing between the two planes of the web and segmented the web as one. It is worth noting that the results did not change when the angular threshold and neighbourhood size were respectively set to 5° and 7.5 mm. Our method correctly differentiated the two planar façades of the web. In this example, the computation time for ours and Rabbani’s methods were 426, and 288 s respectively.

## 6. Conclusions

The use of TLS for construction site progress monitoring and structural dimension compliance control is evolving markedly. However, point clouds collected in complex and dynamic environments such as a construction site are contaminated with outliers. A comprehensive review of current planar and linear segmentation methods suggested that most methods rely on subjective predefined thresholds to group together points with similar attributes, which may result in over- or under-segmentation of the point cloud depending on the data and threshold used. This led to the development of the robust complete linkage segmentation algorithm to introduce a point cloud segmentation method that provides robust results independent from a subjectively predefined and scene-dependent similarity threshold.

The applicability of the newly developed robust complete linkage planar and linear segmentation method was investigated in eight datasets, seven of which were acquired from actual construction site environments. It was demonstrated that the proposed method achieved an overall accuracy of better than 92.5% for all presented datasets, indicating its generic applicability for processing contaminated point clouds acquired from environments such as construction sites.

The segmentation results of two sample subsets of the point clouds were also compared to the region growing method proposed by Rabbani et al. [47]. It was shown that Rabbani’s region growing method resulted in under-segmentation of the rebars and the web of H-section elements, whereas our method was effectively capable of differentiating between different rebar elements and both planar sides of the H-section’s web.

It is important to mention that the size of neighbourhood and the point sampling density may affect the robust classification and consequently the segmentation results. The impact of large registration errors was also not investigated (partly due to the high registration precision of the acquired data), which might adversely affect the segmentation results. In such circumstances, it might be attractive to perform the presented algorithms for robust planar and linear segmentation on a single scan basis, and merge the results while incorporating registration inaccuracies. Furthermore, in the developed system, the impact of random measurement errors on the principal components (eigenvalues and eigenvectors) were estimated based on Monte Carlo simulation of point clouds from a significant number of randomly oriented planes/lines subjected to random instrumental measurement errors. Monte Carlo simulation was adopted since currently an analytical solution to the impact of correlated random measurement errors on the principal components does not exist. Therefore, the development of a closed-form solution to the problem is an interesting topic for future research. Currently, a new method is under development to address the aforementioned limitations.

## Figures and Tables

**Table 1:** # of scan-stations, average # of targets per scan-station and registration precision of the all datasets.

Experiment	Epoch	# of Scan-Stations	Total # of Points (millions)	Average # of Targets per Scan Location	Registration Precision (mm)
Experiment 1: MML	1	3	30	4	1.2
Experiment 2: GSHR	1	3	37	7	1.5
	2	3	153	6	1.4
	3	4	201	8	2.2
	4	3	115	8	1.5
	5	5	358	6	1.8
	6	3	128	7	1.2
Experiment 3: TITL	1	6	537	5	1.7

**Table 2:** Summary of the planar and linear classification and segmentation results.

	**Planar Classification Results**			**Linear Classification Results**			**Overall**		
	**Precision**	**Recall**	**Accuracy**	**Precision**	**Recall**	**Accuracy**	**Precision**	**Recall**	**Accuracy**
Epoch 1	95.0	94.6	91.1	92.6	91.6	98.4	94.7	94.3	94.7
									
Epoch 2	95.4	93.9	91.0	90.9	90.9	97.7	94.7	93.5	94.6
Epoch 3	93.7	95.2	91.1	93.1	92.2	98.6	93.7	94.9	94.8
Epoch 4	94.5	96.4	92.7	90.1	94.1	97.8	93.7	96.0	95.4
Epoch 5	96.5	94.3	92.1	93.0	93.4	98.5	96.0	94.2	95.5
Epoch 6	94.5	93.0	92.3	92.5	91.0	95.1	93.9	92.4	93.7
Overall	95.1	94.8	91.8	91.7	92.4	97.8	94.6	94.4	95.0
	**Planar Segmentation Results**			**Linear Segmentation Results**			**Overall**		
	**Precision**	**Recall**	**Accuracy**	**Precision**	**Recall**	**Accuracy**	**Precision**	**Recall**	**Accuracy**
Epoch 1	97.6	98.1	96.0	95.7	97.3	93.7	97.4	98.1	95.8
Epoch 2	98.3	99.7	98.1	96.1	99.3	95.7	98.0	99.6	97.7
Epoch 3	97.2	98.1	95.6	95.4	95.6	92.0	97.0	97.9	95.2
Epoch 4	96.4	97.1	93.9	97.1	98.9	96.2	96.5	97.4	94.3
Epoch 5	98.1	99.8	98.0	95.8	99.7	95.7	97.8	99.8	97.7
Epoch 6	96.9	97.2	94.7	94.9	98.5	93.9	96.2	97.6	94.4
Overall	97.5	98.5	96.2	96.0	98.7	95.0	97.2	98.6	96.1
